# Ensemble of machine learning algorithms using the stacked generalization approach to estimate the warfarin dose

**DOI:** 10.1371/journal.pone.0205872

**Published:** 2018-10-19

**Authors:** Zhiyuan Ma, Ping Wang, Zehui Gao, Ruobing Wang, Koroush Khalighi

**Affiliations:** 1 Easton Cardiovascular Associates, Easton, PA, United States of America; 2 Division of Cardiology, Department of Medicine, Duke University Medical Center, Durham, NC, United States of America; 3 Department of Mathematics and Statistics, San Diego State University, La Mesa, CA, United States of America; 4 Department of Chemistry and Social Science Research Institute, Duke University, Durham, NC, United States of America; 5 Science Center of Opera Solutions LLC, San Diego, CA, United States of America; 6 Division of Cardiology, Department of Medicine, Easton Hospital, Easton, PA, United States of America; 7 Drexel University College of Medicine, Philadelphia, PA, United States of America; Wroclaw University of Science and Technology, POLAND

## Abstract

Warfarin dosing remains challenging due to narrow therapeutic index and highly individual variability. Incorrect warfarin dosing is associated with devastating adverse events. Remarkable efforts have been made to develop the machine learning based warfarin dosing algorithms incorporating clinical factors and genetic variants such as polymorphisms in *CYP2C9* and *VKORC1*. The most widely validated pharmacogenetic algorithm is the IWPC algorithm based on multivariate linear regression (MLR). However, with only a single algorithm, the prediction performance may reach an upper limit even with optimal parameters. Here, we present novel algorithms using stacked generalization frameworks to estimate the warfarin dose, within which different types of machine learning algorithms function together through a meta-machine learning model to maximize the prediction accuracy. Compared to the IWPC-derived MLR algorithm, Stack 1 and 2 based on stacked generalization frameworks performed significantly better overall. Subgroup analysis revealed that the mean of the percentage of patients whose predicted dose of warfarin within 20% of the actual stable therapeutic dose (mean percentage within 20%) for Stack 1 was improved by 12.7% (from 42.47% to 47.86%) in Asians and by 13.5% (from 22.08% to 25.05%) in the low-dose group compared to that for MLR, respectively. These data suggest that our algorithms would especially benefit patients requiring low warfarin maintenance dose, as subtle changes in warfarin dose could lead to adverse clinical events (thrombosis or bleeding) in patients with low dose. Our study offers novel pharmacogenetic algorithms for clinical trials and practice.

## Introduction

Warfarin is the most widely used oral anticoagulant worldwide. Due to its narrow therapeutic window and large interpatient variability, warfarin dosing remains challenging [1]. The consequence of incorrect warfarin dosing can be devastating, predisposing the patient to thrombosis in the case of under-dosing or bleeding in the case of overdosing. Because of these challenges associated with warfarin use, it is one of the leading causes in emergency department visits and the most often cited cause of drug-related mortality [2]. Clinical factors, demographic variables and genetic variants significantly contribute to interpatient variability in dose requirement for warfarin. While non-genetic factors, such as age, height, weight, race and drug interaction, have been reported to explain 15–20% of the variability in dose, polymorphisms in cytochrome p450, family 2, subfamily C, polypeptide 9 (*CYP2C9*), and vitamin K epoxide reductase complex, subunit1 (*VKORC1*) independently correlate with warfarin therapeutic dose [3–5], and combined polymorphisms of those two genes account approximately 30% (20%-25% for *VKORC1* rs9923231; 5%-10% for *CYP2C9*) of the interpatient warfarin dose variability [3–6].

Remarkable efforts have been made to estimate the appropriate warfarin dose to improve the patient care. Many pharmacogenetic algorithms integrating clinical, demographic and genetic variables have been created to predict the dose requirement in individual patients [7–11]. Most of the dosing algorithms are based on multivariate linear regression (MLR). One of the most widely used and tested algorithms is the IWPC pharmacogenetic algorithm [8], which has proved accuracy in multiple studies. Other advanced machine learning approaches such as deep learning (neural networks), tree-based algorithms and support vector machines have also been used to predict warfarin dose [7, 10, 11], but those studies use a single machine learning algorithm to maximize the accuracy of predicting warfarin dose. In a mathematical point of view, a machine learning algorithm is a sophisticated fit to a non-linear function, and a single machine learning model may fit well to a certain subset of patients, but may overfit or underfit to the rest of patients with different genetic and racial background. As a result, the prediction accuracy of a single model may reach an upper limit even with optimal parameters. It is not surprising that the models produced by MLR and support vector regression with a linear kernel were statistically indistinguishable and significantly outperformed all the other approaches in the IWPC cohort [8]. One way to overcome the limitation of a single algorithm is to combine the advantages of several algorithms to break through the upper limit of a single machine learning algorithm (i.e. ensemble method). Recently, the ensemble method “bagging” has been applied to predict warfarin dose [12, 13]. Stacked generalization is another ensemble method that uses a higher-level model to combine lower-level models to achieve higher prediction accuracy [14, 15]. Unlike the “bagging” and “boosting” approaches which can only combine machine learning algorithms of the same type, stacked generalization can combine different types of algorithms through a meta-machine learning model to maximize the generalization accuracy.

In this study, we created novel regression models to estimate warfarin stable dose utilizing stacked generalization frameworks that combine the advantages of distinct machine learning algorithms and significantly improved the prediction accuracy compared to MLR.

## Materials and methods

### The International Warfarin Pharmacogenetic Consortium (IWPC) Cohort

IWPC Cohort has been described previously [8]. Expanded Data set was downloaded from the PharmGKB website (http://www.pharmgkb.org/downloads/), which contains pooled data on 6256 chronic warfarin users recruited through collaborative efforts of 22 research groups from 4 continents. This data set includes detailed de-identified curated data on demographic factors, clinical features, such as age, weight, height and concomitant use of amiodarone, as well as *CYP2C9* and *VKORC1* genotypes. Missing values for height and weight were imputed with multivariate linear regression models. Specifically, weight, race, and sex were used for the imputation of the height variable, while height, race, and sex were used for the weight variable. For missing values of the *VKORC1* rs9923231, the imputation strategy has been described [8], which is based on linkage disequilibrium in *VKORC1* and race. We excluded 17 subjects with CYP2C9*5, *6, *11, *13 and *14, due to low allele frequency and an outlier subject with warfarin stable dose 315 mg/week. A total of 5743 subjects were included in this study.

### Stacked generalization framework

Predicting warfarin maintenance dose is a regression task, which requires a function or model *f* that maps an input vector x ∈ Ρ^d^ onto the corresponding continuous label value y ∈ Ρ. Since a training data set {(***x***_***1***_, ***y***_***1***_), …, (***x***_***n***_, ***y***_***n***_)} is utilized to find *f*, the task falls into the category of a supervised learning problem. The machine learning problem is formulated as a minimization problem of the form:

$$\underset{f}{\min}\sum_{i=1}^{n}l(f(x_{i}),y_{i})+\mathrm{\lambda r}(f)$$ (1)

The first term of the objective is the empirical risk described by a loss function S which measures the quality of the function f. A specific case is the squared loss *l*(*ŷ*, *y*) = (*ŷ - y*)^2^. The second term of the objective in Eq (1) is a regularization term which measures the complexity or roughness of the function f, which usually is a norm of *f* or its derivatives. L_2_ regularization was used in this study.

Stacked generalization is utilized to ensemble different machine learning algorithms, which can be viewed as a means of collectively using several models to estimate their own generalizing biases with respect to a particular learning set, and then filter out those biases [16, 17]. There are two kinds of models in a stacked generalization framework: several base models (level-0 models) and one meta-model (level-1 model). The essence of stacked generalization is to use the level-1 model to learn from the predictions of level-0 models. Generally, a stacked generalization framework can obtain more accurate prediction compared to the best level-0 model [16].

One of the key points is to obtain the training data for level-1 model (***D***_***cv***_) from cross-validation technique. Given an original data set ***D*** = {(***y***_***n***_, ***x***_***n***_), *n* = 1, …, N}, where *y*_*n*_ is the target value and *x*_*n*_ represents feature vectors of the *n*th instance, randomly split the data into *K* almost equal folds ***D***_***1***_, ***D***_***2***_, …, ***D***_***K***_ (*K* = 5 in this study). Define ***D***_***k***_ and ***D***^**(-k)**^ = ***D***–***D***_***k***_ to be the test and training sets for the *k*_th_ fold of a *K*-fold cross-validation. Given J different level-0 machine learning algorithms (***M***_***1***_, ***M***_***2***_, …, ***M***_***J***_), each ***M***_***j***_ is trained by ***D***^***(-k)***^ and predict each instance ***x*** in ***D***_***k***_. Let ***v***_***k***_^**(-j)**^(***x***) donate the prediction of the model ***M***_***j***_ on ***x***. Then we have:

$$z_{kn}=v_{k}^{\left(-j\right)}(x_{n})$$ (2)

At the end of the entire cross-validation process of each ***M***_***j***_, the data assembled from the outputs of the J models is

$$D_{cv}=\{(y_{n},z_{1n},\ldots ,z_{Jn}),n=1,2,\ldots ,N\}.$$ (3)

***D***_***cv***_ is the training set of level-1 model ***M***_***meta***_. To complete the training process, level-0 models ***M***_***j***_ (j = 1, 2, …, J) are trained using original dataset ***D***, and ***M***_***meta***_ is trained by ***D***_***cv***_.

Now we consider the prediction process, which uses the models ***M***_***j***_, j = 1, 2, …, J, in conjunction with ***M***_***meta***_. Given a new instance, models ***M***_***j***_ produce a vector (***z***_***1***_, …, ***z***_***J***_). This vector is input to the level-1 model ***M***_***meta***_, whose output is the final prediction result for that instance.

### Implementation of machine learning algorithms and parameters

The neural networks (NN), ridge regression (RR), random forest (RF), and extremely randomized trees (ET), support vector regression (SV) were implemented using python library Scikit-learn (version 0.19.1) [18]. Gradient boosting trees (GBT) was implemented using Microsoft’s software ‘LightGBM’ with a python wrapper [19]. Below are the key parameters for each single model: (i) NN layer: 3, hidden layer size: 100, activation function: ‘logistic’, solver: 'lbfgs'; (ii) RR regularization: 1.0; (iii) RF number of trees: 100, maximum depth of the tree: 100; (iv) ET number of trees: 100, maximum depth of the tree: 100; (v) GBT number of trees: 100, Maximum depth of the tree: -1, Learning rate: 0.1; (vi) SV kernel: linear, cache_size: 1000.

In this study, 80% of the eligible patients were randomly chosen as the training set (N = 4594), which was used to train the machine learning models. The rest 20% of the patients (N = 1149) were utilized as the hold-out test set. Various models were then built on the training set and the hold-out test set was used to evaluate the model performance by two metrics. The mean absolute error (MAE), the absolute difference between the predicted and actual maintenance doses, was used to evaluate each model’s predictive accuracy. The mean of the percentage of patients whose predicted dose of warfarin within 20% of the actual stable therapeutic dose (mean percentage within 20%) was utilized to evaluate the clinical significance of each algorithm, as a difference in warfarin dose greater than 20% is likely to be considered to clinically relevant by clinicians. The features/variables in single models were identified based on reported IWPC pharmacogenetic dosing algorithm [8], including height, weight, race, age, enzyme inducer and use of amiodarone. Additional features used in other warfarin stable dose prediction algorithms [7, 9, 10, 20] such as diabetes mellitus, heart failure, valve replacement, smoking, use of statins, smoking status, and other *VKORC1* genotypes (S1 Table) were also included to train the stacked generalization models.

### Warfarin dose subgroup analysis

To assess the performance of the algorithms in different dose ranges, warfarin stable dose was divided into three subgroups based on the 25% and 75% quantiles according to race: low dose (< 15.0 mg/week), intermediate dose (15.0–28.0 mg/week), and high dose (> 28.0 mg/week) in Asians; low dose (< 30.0 mg/week), intermediate dose (30.0–52.5 mg/week), and high dose (> 52.5 mg/week) in blacks; low dose (< 22.0 mg/week), intermediate dose (22.0–42.5 mg/week), and high dose (> 42.5 mg/week) in whites; low dose (< 22.0 mg/week), intermediate dose (22.0–40.0 mg/week), and high dose (> 40.0 mg/week) in the missing or mixed race.

### Statistical analysis

Due to the skewed (with a longer tail at high doses) distribution of warfarin dose, we transformed raw dose into square root of the dose. To obtain robust statistics, 100 rounds of resampling were performed from the IWPC cohort. All the quantitative data are presented as means with 95% confidence intervals (CI). P values < 0.05 were considered to be statistically significant. The MAE and differences of the mean percentage within 20% of the actual stable dose among the algorithms were compared with unpaired Student’s t-test. All statistical analyses were conducted with R (version 3.4.4).

## Results

### Basic characteristics of the IWPC cohort

The characteristics of the patients are shown in Table 1 and S1 Table. In the IWPC cohort, 5743 patients were included for analyses with a median warfarin stable dose of 28.0 mg/week. The target range for International Normalized Ratio (INR) fell within 1.7 to 3.3, with the majority of being prespecified between 2 and 3. The proportions of patient age less than 50, 50 to 80, and older than 80 were 17.0%, 71.0%, and 12.1%, respectively. There were 4.9% of patients concomitantly taking amiodarone, 19.6% of patients taking statins, 1.1% of patients using enzyme inducers (including phenytoin, carbamazepine, and rifampin). There were 8.4% of patients smoking. Patients with diabetes mellitus, cardiomyopathies and heart failure, and valve replacement were 10.8%, 12.5% and 17.0%, respectively.

**Table 1:** Demographic and clinical characteristics of the IWPC cohort.

Variable	IWPC data (n = 5743)
Warfarin dose—mg/week	
Mean (SD)	32.0 (16.8)
Median	28.0
Interquartile range	20.0–40.0
Genotype—no. (%)	
*VKORC1* rs9923231	
G/G	1887 (32.9)
A/G	2065 (36.0)
A/A	1683 (29.3)
Unknown	108 (1.9)
*CYP2C9*	
*1/*1	4232 (73.7)
*1/*2	755 (13.2)
*1/*3	482 (8.4)
*2/*2	58 (1.0)
*2/*3	68 (1.2)
*3/*3	20 (0.4)
Unknown	128 (2.2)
Age—no. (%)	
< 50	974 (17.0)
50–80	4075 (71.0)
> 80	694 (12.1)
Height—m	
Median	167.6
Interquartile range	160.0–175.8
Weight—kg	
Median	76.0
Interquartile range	63.0–90.7
Race—no. (%)	
White	3095 (53.9)
Asian	1517 (26.4)
Black	665 (11.6)
Mixed or missing	466 (8.1)
Enzyme inducer	61 (1.1)
Amiodarone	280 (4.9)
Statin	972 (16.9)
Smoker	482 (8.4)
DM	619 (10.8)
Heart failure	716 (12.5)
Valve replacement	975 (17.0)

### Overall performance of the single algorithms

To establish the stacked generation frameworks to ensemble different models, we first examined the predictive performance of several single models. Eight different machine learning algorithms including MLR, SV, RR, NN, GBT, RF, ET, and K nearest neighbors (KN) were trained by the training set and then prediction was made on the hold-out set. The performance of each model measured by the MAE and percentage within 20% is shown in Table 2. The algorithms SV, RR, and MLR performed statistically indistinguishable and were the best among eight algorithms, followed by GBT and NN. The algorithms RF, ET and KN resulted in the least favorable results.

**Table 2:** Comparison of the performance of individual machine learning algorithms.

Algorithms	MAE (95% CI)	Within 20% (95% CI)	P value (vs. MLR)
**MLR**	8.53 (8.08–8.99)	46.31 (43.73–48.89)	
**SV**	8.52 (8.11–8.93)	46.51 (44.08–48.97)	0.639*; 0.279^#^
**RR**	8.52 (8.12–8.92)	46.29 (43.74–48.84)	0.758*; 0.907^#^
**NN**	8.84 (8.35–9.33)	44.35 (41.50–47.20)	<0.001*; <0.001^#^
**GBT**	8.82 (8.42–9.23)	44.88 (42.47–47.29)	<0.001*; <0.001^#^
**RF**	9.28 (8.84–9.73)	42.88 (40.16–45.59)	<0.001*; <0.001^#^
**ET**	10.18 (9.73–10.63)	39.02 (36.81–41.22)	<0.001*; <0.001^#^
**KN**	10.86 (10.32–11.40)	36.43 (33.89–38.96)	<0.001*; <0.001^#^

* P value for MAE

# P value for Within 20%

Within 20%: the mean of the percentage of patients whose predicted dose of warfarin within 20% of the actual stable therapeutic dose.

SV: Support Vector Machine; RR: Ridge Regression; MLR: Multivariate Linear Regression; NN: Neural Network; GBT: Light Gradient Boosting Machine; RF: Random Forests; ET: Extremely Randomized Tree; KN: K nearest neighbors.

### Configuration and performance of stacked generalization framework 1 and 2 (Stack 1 and 2)

**Fig 1:**
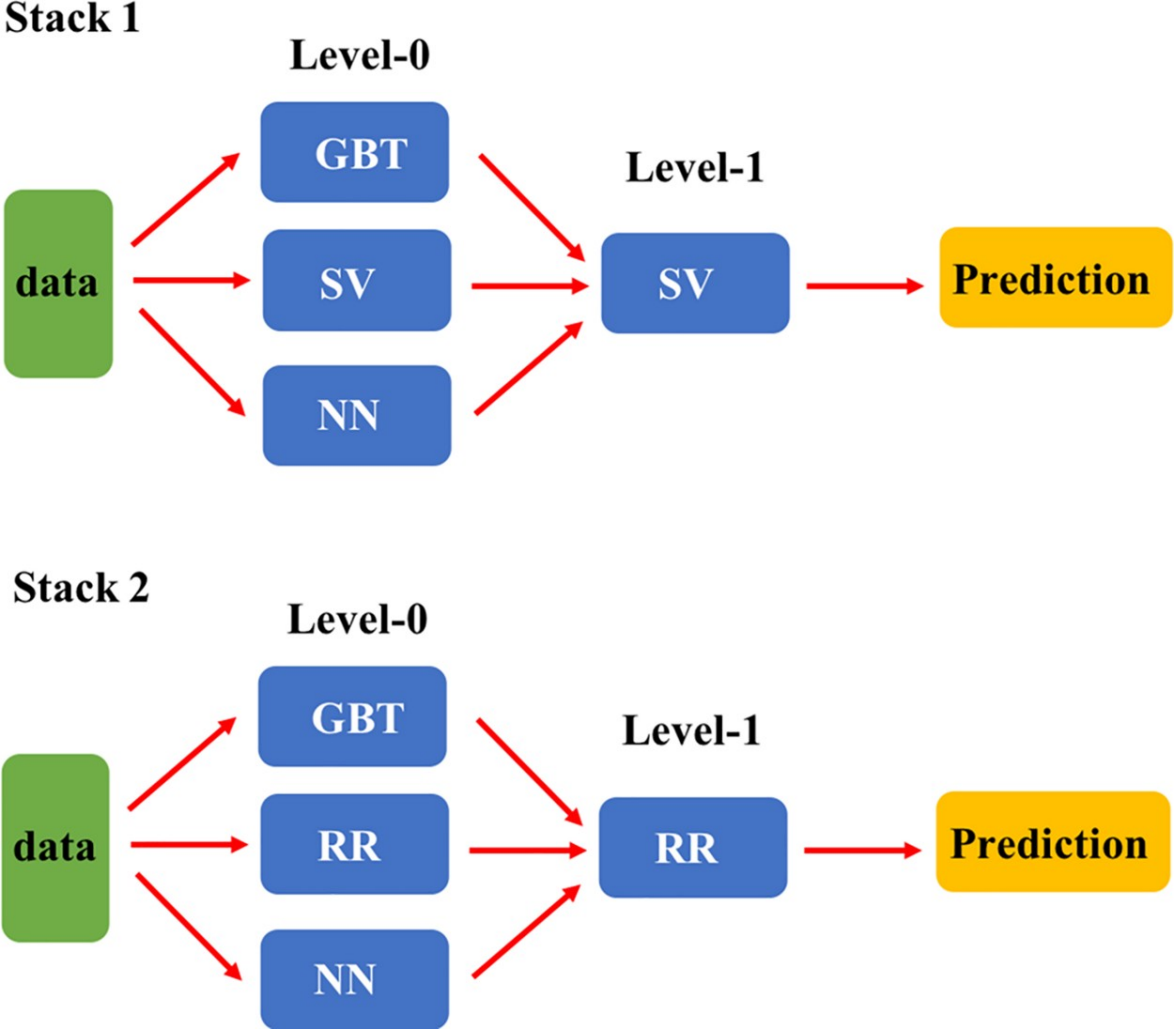
Schematic representation of the configurations of the stacked generalization frameworks (Stack 1 and 2) built from base models. SV: Support Vector Regression; RR: Ridge Regression; NN: Neural Network; GBT: Light Gradient Boosting Machine.

To determine whether ensemble predictors constructed using stacked generalization improve the prediction accuracy for warfarin stable dose, we constructed two different stacked generalization frameworks Stack 1 and 2 using the exactly same parameters in individual algorithms. The frameworks in this study consisted of three level-0 models and one level-1 model (meta model). Fig 1 exhibits the detailed configuration of each framework. The individual algorithms (SV, RD, NN and GBT) in the frameworks were chosen based on the type of the algorithm (i.e. linear regression, neural network and tree-based) and individual predictive performance in Table 2. To improve the prediction accuracy, we included diabetes mellitus, heart failure, valve replacement, smoking, use of statins, and other *VKORC1* genotypes as additional features to train the frameworks (Stack1 and 2). The MAEs for the Stack1 and 2 were 8.31 and 8.31 mg/week, which were significantly better than 8.53 mg/week in the MLR (Table 3). The mean percentage within 20% produced by Stack 1 and 2 (47.85% and 47.81%) were significantly higher than that (46.31%) of MLR (Table 3).

**Table 3:** Overall comparison of the performance of the Stack 1 and 2 with MLR.

Algorithms	MAE (95% CI)	Within 20% (95% CI)	P value (vs. MLR)
**Stack 1**	8.31 (7.86–8.76)	47.85 (45.43–50.28)	<0.001*; <0.001^#^
**Stack 2**	8.31 (7.87–8.76)	47.81 (45.44–50.19)	<0.001*; <0.001^#^
**MLR**	8.53 (8.08–8.99)	46.31 (43.73–48.89)	

* P value for MAE

# P value for Within 20%

Within 20%: the mean of the percentage of patients whose predicted dose of warfarin within 20% of the actual stable therapeutic dose.

### Evaluating the performance of the stacks by race

Given that genetic diversity is known to be great in different races [21], to further examine the performance of the Stacks in different races, Stack 1 and Stack 2 were evaluated in Asians, whites and blacks. The MAE of prediction was highest in blacks and lowest in Asians across the three algorithms (Table 4). In Asians, Stack 1 and Stack 2 performed significantly better than MLR for both MAE and mean percentage within 20% (Table 4). The mean percentage within 20% for Stack 1 and 2 had a 12.7% improvement (from 42.47% to 47.86% and 47.66%) compared to that for MLR. In whites, the MAEs for Stack 1 and 2 were lower than that of MLR (P = 0.002). The performance of Stack 1 and Stack2 in blacks was better than MLR, but there was no statistical significance (Table 4).

**Table 4:** Comparison of the performance of the Stack 1 and 2 with MLR in Asians, whites and blacks.

Algorithms	Asian		White		Black	
	MAE (95% CI)	Within 20% (95% CI)	MAE (95% CI)	Within 20% (95% CI)	MAE (95% CI)	Within 20% (95% CI)
**Stack 1**	6.13 (5.58–6.68)	47.86 (42.29–53.42)	8.70 (8.04–9.36)	48.43 (44.77–52.10)	11.88 (10.13–17.64)	44.90 (37.09–52.73)
**Stack 2**	6.14 (5.60–6.69)	47.66 (42.22–53.10)	8.70 (8.05–9.35)	48.46 (44.70–52.21)	11.92 (10.17–13.66)	44.73 (36.32–53.13)
**MLR**	6.64 (6.12–7.16)	42.47 (38.01–46.93)	8.85 (8.17–9.53)	48.13 (44.67–51.60)	12.00 (10.32–13.68)	43.97 (36.49–51.44)
**P value**	<0.001*; <0.001^#^	<0.001*; <0.001^#^	0.002*; 0.002^#^	0.244*; 0.209^#^	0.360*; 0.518^#^	0.093*; 0.187^#^

* P value for Stack1 vs. MLR

# P value for Stack2 vs. MLR

Within 20%: the mean of the percentage of patients whose predicted dose of warfarin within 20% of the actual stable therapeutic dose.

### Evaluating the performance of the stacks by warfarin dose range

We next to test the predictive ability of Stack 1 and 2 in three different warfarin dose ranges. The MAE of prediction was highest in high dose and lowest in intermediate dose across the three algorithms (Table 5). In the low-dose group, Stack 1 and Stack 2 provided a significantly better prediction of dose than MLR for both MAE and mean percentage within 20% (Table 5). The mean percentage within 20% for Stack 1 was improved by 13.5% (from 22.08% to 25.05%) compared to that for MLR. Likewise, in the intermediate-dose group, Stack 1 and Stack 2 performed significantly better than MLR for both MAE and mean percentage within 20% (P < 0.001). The mean percentage within 20% for Stack 1 and 2 had an about 2.0% improvement (from 60.90% to 62.13% and 61.96%) compared with that for MLR. In the high-dose groups, the mean percentage within 20% for Stack 2 was improved by 3.0% (from 41.11% to 42.35%) compared to that for MLR (P < 0.001).

**Table 5:** Comparison of the Stack 1 and 2 with MLR in three dose ranges.

Algorithms	Low dose		Intermediate dose		High dose	
	MAE (95% CI)	Within 20% (95% CI)	MAE (95% CI)	Within 20% (95% CI)	MAE (95% CI)	Within 20% (95% CI)
**Stack 1**	8.11 (7.37–8.87)	25.05 (19.85–30.25)	5.47 (5.09–5.84)	62.13 (58.32–65.94)	14.43 (13.06–15.80)	41.75 (36.42–47.10)
**Stack 2**	8.27 (7.50–9.03)	24.66 (19.60–29.73)	5.50 (5.13–5.88)	61.96 (58.15–65.78)	14.23 (12.83–15.64)	42.35 (36.80–47.89)
**MLR**	8.62 (7.91–9.33)	22.08 (17.88–26.29)	5.59 (5.22–5.95)	60.90 (56.84–64.95)	14.61 (13.28–15.94)	41.11 (36.80–45.24)
**P value**	<0.001*; <0.001^#^	<0.001*; <0.001^#^	<0.001*; <0.001^#^	<0.001*; <0.001^#^	0.072*; <0.001^#^	0.071*; <0.001^#^

* P value for Stack1 vs. MLR

# P value for Stack2 vs. MLR

Within 20%: the mean of the percentage of patients whose predicted dose of warfarin within 20% of the actual stable therapeutic dose.

## Discussion

The individual response to warfarin is highly variable, being influenced by clinical factors and genetic variants such as polymorphisms in *CYP2C9* and *VKORC1*. Given that incorrect warfarin dosing can potentially increase the risk of thrombosis or bleeding, estimating appropriate warfarin dose for individual patient has been an active research area. Recently, personalized genotype-guided warfarin dosing has demonstrated clinical benefits and superior clinical outcomes in major clinical trials [22–24]. In these trials, predicted warfarin maintenance doses were based on MLR. However, due to the complex and non-linear association between warfarin dose and clinical and genetic variables, such limitations related to MLR-based algorithms may affect the prediction accuracy. Because of that, in this study, we set out to develop novel algorithms using the stacked generalization approach to estimate the warfarin dose, where different types of machine learning algorithms function together through a meta-machine learning model to maximize the prediction accuracy. This strategy has been successfully applied to and significantly improved the prediction of molecular atomization energies [25].

To be able to directly compare the performance among different algorithms, we used the IWPC data set and applied this data set to eight different machine learning algorithms with the same covariates in the IWPC algorithm. In line with the MAE (8.5 mg/week, 95% CI (8.0–9.0)) in the IWPC study [8], the MAE for MLR in this study was 8.53 mg/week (95% CI (8.08–8.99)). These data validated our data processing and allowed us to directly compare the performance of algorithms developed in this study to the IWPC algorithms. Moreover, we found that MLR provided a comparable performance as SV and RR and outperformed all other algorithms, including NN and GBT, which is consistent with the results reported in the IWPC study [8]. In order to take advantage of different algorithms, we constructed ensemble predictor Stack 1 and 2 using SV, RR, NN and GBT. Overall, Stack 1 and 2 performed significantly better than MLR. Subgroup analysis revealed that the mean percentage within 20% for Stack 1 and 2 had a 12.7% improvement (from 42.47% to 47.86% and 47.66%) compared to that for MLR in Asians. Similarly, the mean percentage within 20% for Stack 1 was improved by 13.5% (from 22.08% to 25.05%) compared to that for MLR in the low-dose group. The median warfarin weekly dose for Asians was lower compared to whites and blacks [21]. These data suggest that our algorithm would especially benefit patients requiring low warfarin dose, as subtle changes in warfarin dose could result in adverse clinical events such as thrombosis or bleeding.

Many studies developed the pharmacogenetic algorithms for warfarin dosing [7–11], but the performance of these algorithms can hardly be compared due to different data sets with different patient ethnicity and prevalence of *CYP2C9* and *VKORC1* polymorphisms. For example, we have reported that Asians, whites and blacks have different warfarin sensitivity, resulting from different polymorphisms of *CYP2C9* and *VKORC1* in the IWPC cohort [21]. For the IWPC data set, additional algorithms such as multivariate adaptive regression splines (MARS), lasso regression (LAR) and Bayesian additive regression trees (BART) have also been tested along with MLR [10]. Interestingly, the MAE for MLR was 9.28 mg/week in that study and they showed that BART, MARS and SVR performed superior than MLR in the prediction of warfarin dose. However, MLR and SVR performed better than MARS in the IWPC study. This discrepancy could be owing to different data processing and independent variables selected. In addition, the ensemble method “bagging” has been used to predict warfarin dose [12, 13], which is a popular method to assemble base models to decrease the variance, but not to improve the predictive force. Therefore, in terms of the coefficient of determination (*R*^*2*^), *R*^*2*^ for the IWPC algorithm in Asians is 0.46 [26], which is not inferior to the “bagging” models (Ms+G) ranging from 0.402 to 0.441 for Chinese in the previous study [13]. “Bagging” method requires designing various feature functions for base models in order to achieve diversity, which is a key requirement for base models [13]. The novel regression models we proposed here can take advantage of distinct machine learning algorithms to achieve high diversity of base models. Designing feature functions is not necessary and all base models can use the same feature space, which can avoid extra information extraction efforts. Moreover, “voting” has been combined with “bagging” to assemble the base models to predict warfarin dose [12]. In contrast to “stacking”, no learning takes place at the meta-level when combining base models by a voting scheme. Of note, “stacking” has been applied to combine different base models to predict warfarin dosing adjustment based on time-series data with patient’s history of warfarin dose and INR [27], whereas in this study we focused on the development of pharmacogenetic models to predict warfarin maintenance dose using clinical and genetic factors. Taken together, the performance of our algorithms based on the stacked generalization framework appears superior to other published models [10]. This is probably attributable to the power of our model to identify complex, nonlinear relationships among genetic and clinical variables and to deal with many sources of inferential trouble such as outliers and collinearity among variables compared to the MLR in the IWPC study.

Our study has several limitations. First, due to the missing values in the IWPC cohort, we imputed missing genotypes of *VKORC1* for some patients based on linkage disequilibrium [5]. We also imputed missing values for height and weight using multivariate linear regression models. These imputation strategies, which are generally reliable, could have introduced some errors. Second, many patients required very high doses of warfarin in the IWPC cohort (> 70 mg/week), especially in warfarin normal groups. The polymorphisms explored in *VKORC1* and *CYP2C9* to classify warfarin sensitivity primarily explain increased sensitivity to warfarin, not increased resistance. Mutations of *VKORC1* have been associated with resistance to warfarin [28, 29]. Third, our algorithms are more complex than MLR, which may not be very easy to be implemented in clinical setting.

In conclusion, we created novel regression models using the stacked generalization approach to estimate the warfarin maintenance dose. The performance of our algorithms was superior to the algorithm developed by IWPC, especially in Asians and the low-dose group. Our study offers alternative pharmacogenetic algorithms for clinical trials and practice.

## Supporting information

S1 Table The genotypes of *VKORC1* in the IWPC cohort. (DOCX)
