# Supplementary material for: Ensemble of machine learning algorithms using the stacked generalization approach to estimate the warfarin dose
Source: PLoS One. 2018 Oct 19;13(10):e0205872. doi: 10.1371/journal.pone.0205872 (PMC6195267; doi:10.1371/journal.pone.0205872)
Supplement: S1 Table — (DOCX) [file pone.0205872.s001.docx]

S1 Table. The genotypes of *VKORC1* in the IWPC cohort.

| **Variable** | **IWPC data (n=5743)** |
| --- | --- |
| Genotype—no. (%) |  |
| *VKORC1* rs2884737 |  |
| G/G | 93 (1.6) |
| G/T | 629 (11.0) |
| T/T | 1585 (27.6) |
| Unknown | 3436 (59.8) |
| *VKORC1* rs9934438 |  |
| C/C | 1037 (18.1) |
| C/T | 1043 (18.2) |
| T/T | 1431 (24.9) |
| Unknown | 2232 (38.9) |
| *VKORC1* rs8050894 |  |
| C/C | 1287 (22.4) |
| C/G | 1338 (23.3) |
| G/G | 1071 (18.7) |
| Unknown | 2047 (35.6) |
| *VKORC1* rs7294 |  |
| A/A | 390 (6.8) |
| A/G | 1293 (22.5) |
| G/G | 1896 (33.0) |
| Unknown | 2164 (37.7) |
| *VKORC1* rs2359612 |  |
| C/C | 711 (12.4) |
| C/T | 888 (15.5) |
| T/T | 1162 (20.2) |
| Unknown | 2982 (51.9) |
| *VKORC1* rs17880887 |  |
| A/A | 71 (1.2) |
| A/C | 268 (4.7) |
| C/C | 601 (10.5) |
| Unknown | 4803 (8.4) |
